# Pancreas Transplant Training in U.S. Transplant Nephrology Fellowships: National Survey of Current Barriers and Potential Solutions

**DOI:** 10.1111/ctr.70674

**Published:** 2026-09-09

**Authors:** Alissar El Chediak, Ayush Singh, Arjun Singh, Arya Ghiwala, Mital Shah, Sumi Nair, Roy D. Bloom, Michelle A. Josephson, Neeraj Singh

**Affiliations:** ^1^ Division of Nephrology, Department of Internal Medicine UT Southwestern Medical Center Dallas Texas USA; ^2^ Tulane University New Orleans Louisiana USA; ^3^ John P. Stevens High School Edison New Jersey USA; ^4^ Woodbridge Academy Woodbridge New Jersey USA; ^5^ Division of Nephrology, Department of Internal Medicine Robert Wood Johnson University Hospital New Brunswick New Jersey USA; ^6^ Division of Nephrology, Department of Internal Medicine Mayo Clinic Scottsdale Arizona USA; ^7^ Department of Medicine University of Pennsylvania Perelman School of Medicine Philadelphia Pennsylvania USA; ^8^ Division of Nephrology, Department of Internal Medicine University of Chicago Chicago Illinois USA

**Keywords:** pancreas volume, pancreas transplant training, transplant nephrology fellows

## Abstract

Limited training opportunities for transplant nephrologists have been identified as a potential contributing factor for declining pancreas transplant volumes. However, data describing pancreas transplant training within transplant nephrology fellowships are scarce. We conducted a survey of 66 accredited U.S. transplant nephrology fellowship programs between December 2024 and May 2025 to assess perceived barriers and strategies to enhance pancreas transplant training. A total of 48 (72%) responses were received. A significant number of programs (*n* = 21; 44%) reported that they did not graduate fellows to meet the Organ Procurement and Transplantation Network eligibility requirements to become primary pancreas transplant physicians. 14 (29%) programs either did not perform pancreas transplants or performed fewer than 5 annually. Low pancreas transplant volume was identified as the most significant barrier to training. The most frequently endorsed solutions included designing dedicated pancreas transplant rotations, even at institutions outside their own, and implementing a standardized training curriculum. Such interventions may help address current training gaps and strengthen the future pancreas transplant workforce.

**Abbreviations**
AST: American Society of Transplantation
ATC: American Transplant Congress
IRB: Institutional Review Board
OPTN: Organ Procurement and Transplantation Network
REDCap: Research Electronic Data Capture
U.S: United States

## 1 Introduction

Pancreas transplant is an important treatment option for patients with diabetes and kidney failure. Despite improvements in patient and graft survival, the number of pancreas transplants performed nationally has declined [1]. A workshop organized by the American Society of Transplantation (AST) identified barriers to pancreas transplantation [2]. Among the most significant barriers was the perceived lack of adequate training in pancreas transplantation due to declining pancreas transplant volume [2]. Currently, the Organ Procurement and Transplantation Network (OPTN) requires transplant nephrology fellows to be involved in the care of at least 8 newly transplanted pancreas recipients, with a minimum follow‐up of 3 months during the fellowship period [3]. However, it is unclear what proportion of training programs are training fellows to meet OPTN requirements. The objective of this study was to gather insights from transplant nephrology fellowship program directors regarding the current state of pancreas transplant training, identify existing challenges, and explore potential solutions to enhance the training experience for future specialists.

## 2 Methodology

We conducted an email‐based survey of all active transplant nephrology fellowship program directors in the United States (*n* = 66) from December 2024 to May 2025, with an initial email followed by two subsequent email reminders. The survey instrument (S1) was developed by the study investigators and consisted of three sections. Section 1 collected program‐level characteristics, including the number of fellowship positions, unfilled positions, and pancreas transplant volume. Section 2 assessed pancreas transplant training, including the OPTN eligibility to serve as a pancreas transplant medical director, the presence of dedicated pancreas transplant advocates, and available educational activities. Section 3 explored perceived barriers to training and potential solutions, with respondents ranking the relative impact and effectiveness of each. The study was approved by the Institutional Review Board at the University of Texas Southwestern Medical Center (IRB #STU‐2024‐1080).

Study data were collected and managed using REDCap (Research Electronic Data Capture) tools hosted at the University of Texas Southwestern [4, 5]. Incomplete responses were excluded to maintain data integrity. Descriptive statistics, including frequencies and percentages, were calculated for all categorical variables. Respondents ranked various barriers to pancreas transplant training on a scale from 1 (most impactful) to 7 (least impactful), and mean rankings were computed for each reported barrier. Similarly, reported strategies to enhance training effectiveness were ranked using a mean score on a scale from 1 (most impactful) to 7 (least impactful).

## 3 Results

We collected 52 survey responses during the survey period; however, 2 were incomplete, and 2 were duplicates, leaving 48 completed surveys for analysis, corresponding to a response rate of 72%. Among respondents, 29% (*n* = 14) programs performed 0–5 pancreas transplants per year, 19% (*n* = 9) programs performed 6–9 pancreas transplants per year, 33% (*n* = 16) programs performed 10–20 pancreas transplants per year, and 19% (*n* = 9) programs performed more than 20 pancreas transplants per year.

### 3.1 Pancreas Transplant Training: Current State

Approximately 2/3 (*n* = 31) of transplant nephrology programs offered 1 fellowship position per year, while 1/3 (*n* = 17) offered 2 fellowship positions per year during the study period. 1/third (*n* = 16) failed to fill a spot at least once during the past three years, and 1/fourth (*n* = 12) failed to fill a position twice during this period.

Overall, 56% (*n* = 27) of program directors reported that their programs enabled fellows to meet the OPTN requirements for becoming pancreas transplant primary physicians, whereas 44% (21 out of 48) of program directors reported that their programs did not enable graduating fellows to meet OPTN eligibility criteria to become a pancreas transplant program medical director.

Table 1 compares pancreas transplant programs that met OPTN requirements for training fellows to become primary pancreas physicians with those that did not, as reported by program directors. Programs which met current OPTN requirements (*n* = 27) were significantly more likely than those that did not (*n* = 21) to be located in the Upper Midwest or Eastern Seaboard (68.0% vs. 27.8%, *p* = 0.015), perform >10 pancreas transplants annually (85.2% vs. 10.0%, *p* < 0.001), and have dedicated pancreas transplant physicians and/or surgeons (88.9% vs. 66.7%, *p* = 0.046). They also offered more fellowship positions per year (1.52±0.51 vs. 1.15±0.37, *p* = 0.006), were more likely to provide opportunities to observe pancreas procurements and surgeries (78.3% vs. 37.5%, *p* = 0.007) and were rated as providing high‐quality pancreas transplant training (59.3% vs. 5.0%, *p* < 0.001). Both groups demonstrated similarly strong support for a national pancreas transplant curriculum (88.9% vs. 90.5%) and regional training centers of excellence (96.3% vs. 100.0%).

**TABLE 1:** Comparison of pancreas transplant programs that met OPTN requirements for training fellows to become primary pancreas physicians with programs that did not meet these requirements, as reported by program directors.

Survey items	Met current OPTN requirements (*N* = 27)	Did not meet current OPTN requirements (*N* = 21)	*p*‐value
Geographical Location/Region, *n/N* (%)			**0.015**
Upper Midwest ^a^, Eastern Seaboard ^b^	17/25 (68.0%)	5/18 (27.8%)	
West ^c^, Southwest ^d^	2/25 (8.0%)	7/18 (38.9%)	
Other Regions ^e^	6/25 (24.0%)	6/18 (33.3%)	
Annual Pancreas Transplants (SPK, PAK, or PTA) Performed by Centers, *n/N* (%)			
≤5	0/27 (0.0%)	13/20 (65.0%)	**<0.001**
6 to 9	4/27 (14.8%)	5/20 (25.0%)	
10 to 20	14/27 (51.9%)	2/20 (10.0%)	
>20	9/27 (33.3%)	0/20 (0.0%)	
Dedicated Pancreas Transplant Surgeon and/or Physician Availability *n/N* (%)			**0.046**
Yes	24/27 (88.9%)	14/21 (66.7%)	
No	3/27 (11.1%)	7/21 (33.3%)	
Receives Direct Endocrinology Referrals, *n/N* (%)			0.472
Yes	23/27 (85.2%)	16/21 (76.2%)	
No	4/27 (14.8%)	5/21 (23.8%)	
Transplant Fellowship Spots Offered per Year by Program, Mean ± SD (*n* = 27 vs *n* = 20)	1.52 ± 0.51	1.15 ± 0.37	**0.006**
Offers Formal Pancreas Training to Fellows, *n/N* (%)			0.472
Yes	23/27 (85.2%)	16/21 (76.2%)	
No	4/27 (14.8%)	5/21 (23.8%)	
Training offered in Pancreas Transplant Biopsy	4/23 (17.4%)	1/16 (6.3%)	0.619
Opportunity in Observing Pancreas Procurements and Surgeries	18/23 (78.3%)	6/16 (37.5%)	**0.007**
Perceived Level of Pancreas Transplant Fellowship Training*, n/N* (%)			**<0.001**
High Quality (Excellent/Good)	16/27 (59.3%)	1/20 (5.0%)	
Baseline/Substandard (Adequate/Inadequate/Poor)	11/27 (40.7%)	19/20 (95.0%)	
Open to Implementing National Training Curriculum/Guidelines for Pancreas Transplant Training, *n/N* (%)			0.861
Open (Very / Somewhat Open)	24/27 (88.9%)	19/21 (90.5%)	
Neutral or Opposed (Neutral / Not Open)	3/27 (11.1%)	2/21 (9.5%)	
Support in Developing National/Regional Centers of Excellence for Pancreas Transplant Training for Fellowship Rotations, *n/N* (%)			0.999
Supportive (Yes / Maybe)	26/27 (96.3%)	21/21 (100.0%)	
Not Supportive (No)	1/27 (3.7%)	0/21 (0.0%)	

Abbreviations – OPTN, Organ Procurement and Transplantation Network; PAK, Pancreas After Kidney; PTA, Pancreas Transplant Alone; SD, Standard Deviation; SPK, Simultaneous Pancreas‐Kidney.

TM Only the states with training programs that participated in the survey are included in the regions described.

^a^ Upper Midwest: (IL, IN, MI, MN, OH, WI);

^b^ Eastern Seaboard: (CT, MA, NY, PA, MD, DC, VA, NC, SC, GA, FL);

^c^ West: (CA, UT, OR);

^d^ Southwest: (AZ, TX);

^e^ Other Regions: (AL, MO, NE, TN).

Formal pancreas transplant–specific education was offered by 83% (40 out of 48) transplant nephrology fellowship programs, most commonly through lectures (*n* = 38), direct patient care (*n* = 37), and participation in patient evaluation and selection committee meetings (*n* = 36). About 10% (*n* = 5) of programs reported offering formal training in pancreas allograft biopsies. Only 35% (*n* = 17) of program directors rated the adequacy of training in pancreas transplant for fellows at their current level as good or excellent.

### 3.2 Barriers to Dedicated Pancreas Training

**FIGURE 1:**
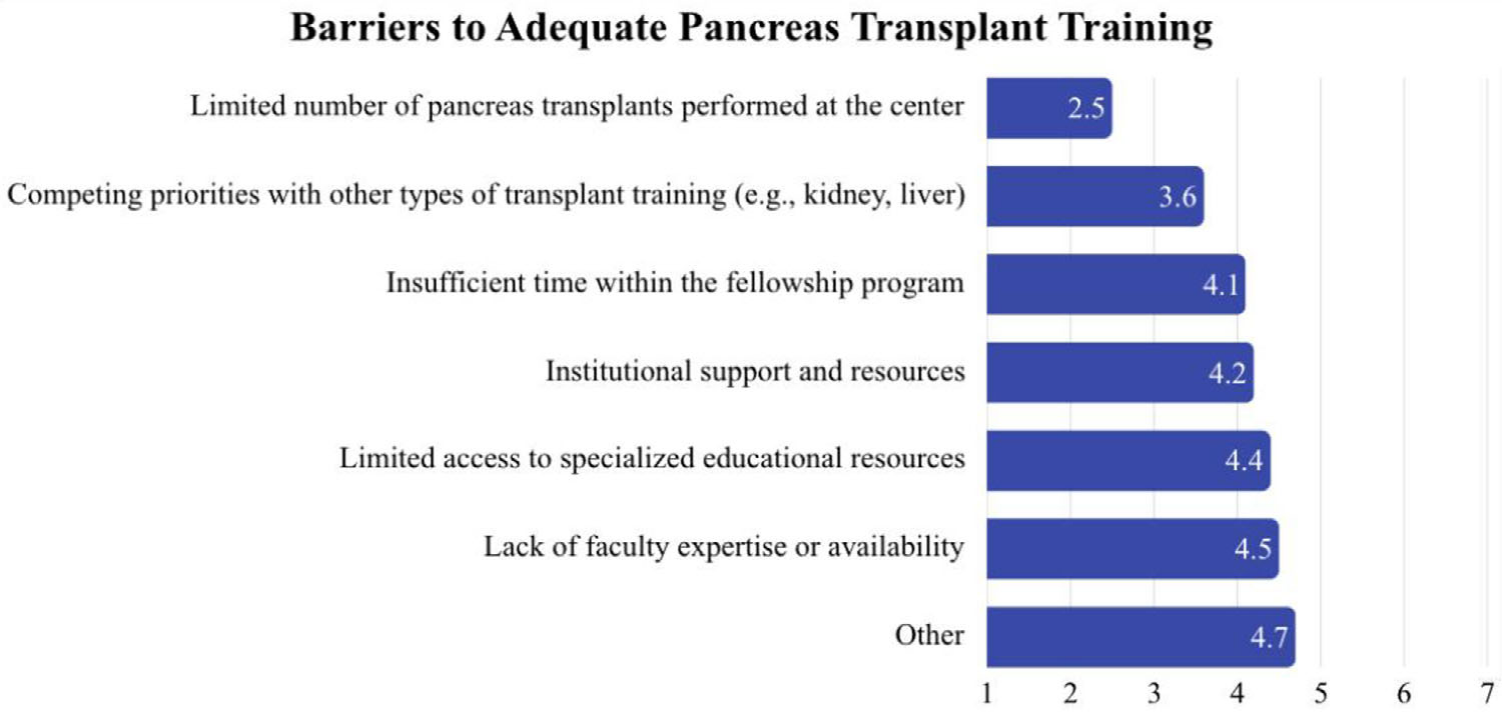
Barriers to adequate pancreas transplant training on a scale from 1 to 7, with 1 being the most impactful and 7 being the least impactful. The responses show the mean score for each barrier across all respondents.

The survey identified various barriers (1 = most impactful to 7 = least impactful) to dedicated pancreas training (Figure 1).

### 3.3 Solutions to Enhance Pancreas Transplant Training

**FIGURE 2:**
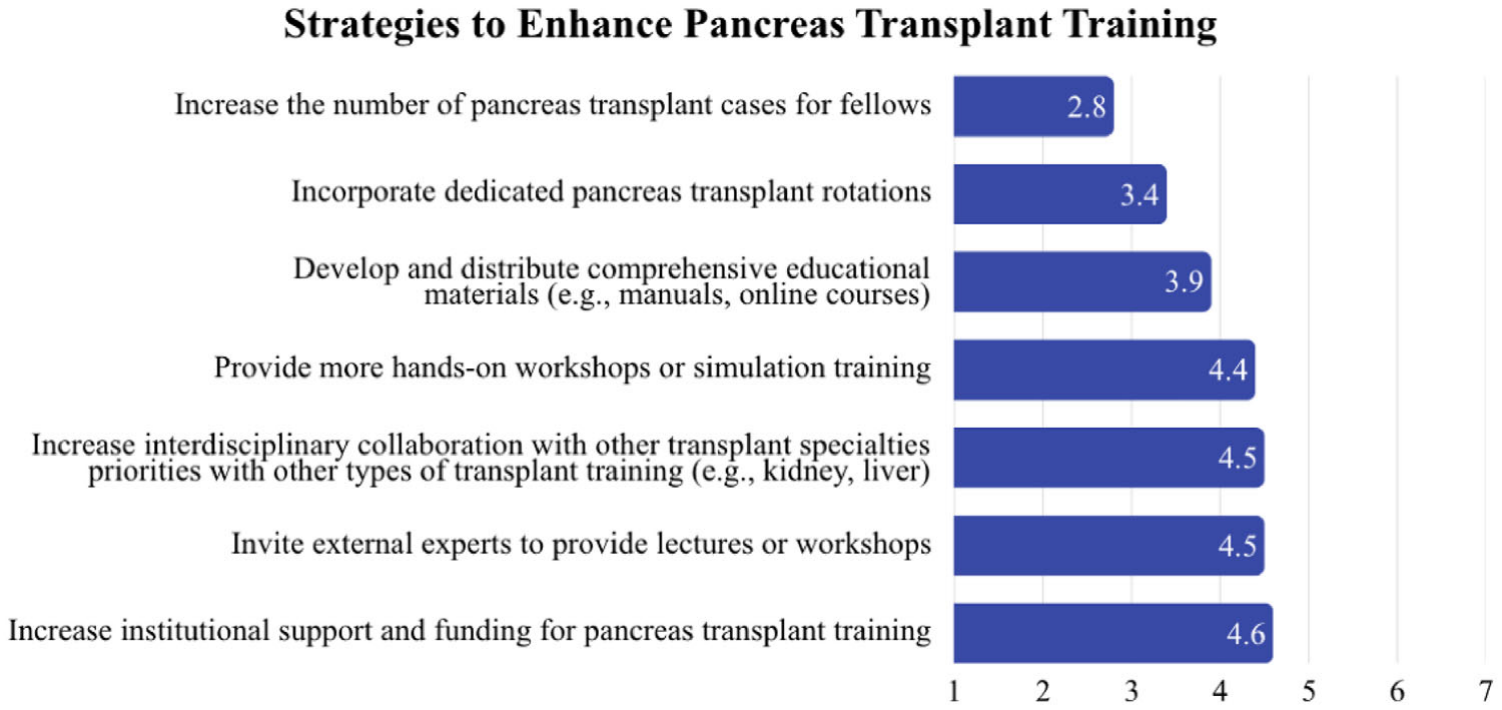
Ranking strategies to enhance pancreas transplantation training on a scale from 1 to 7, with 1 being the most impactful and 7 being the least impactful. The responses show the mean score for each barrier across all respondents.

The survey participants identified strategies for enhancing pancreas transplant training, rated from 1 (most effective) to 7 (least effective) (Figure 2).

Moreover, 67% (*n* = 32) of transplant nephrology program directors reported being very open to implementing a standardized national curriculum or guidelines for pancreas transplant training. In addition, 79% (*n* = 38) indicated support for the development of a pancreas transplant center of excellence, where fellows could rotate to gain additional experience. When asked about the additional resources or support needed to overcome barriers to effective pancreas transplant training at their institutions, respondents provided a wide range of answers (Table 2).

**TABLE 2:** Summary of additional suggestions and resources related to pancreas transplant training and support.

Theme	Description
Educational Resources and Training	Having more formalized rotations and materials
	Need for AST monthly lectures, journal clubs, and education about the topics.
	Shared didactics with transplant surgery. Review pancreas offers with the surgeons.
Long‐term Patient Care Involvement	Advocating a standardized approach to post‐transplant care
	Arrange for fellows to follow pancreas patients in their continuity clinics
Financial and Institutional Support	Increased funding and additional personnel to support pancreas transplantation efforts. Support from Radiology and Pathology for pancreas biopsies.
	Addressing the barriers faced in obtaining medical directorship certification.
Interdisciplinary Collaboration	Involving endocrinologists in the transplant process highlights the need for improved referral systems.
Patient and Community Engagement	Creation of educational materials aimed at patients
	Addressing the challenges associated with insurance for a pancreas transplant.
Fellowship Program Requirements	More transparent descriptions of fellowship programs, to include details about transplant volume and the eligibility criteria necessary to meet OPTN requirements
Hands‐on Experience	Incorporating pancreas procurement and transplantation into the fellowship completion requirements
Exposure to High‐Volume Centers	Providing the opportunity for fellows to visit and learn from high‐volume transplant centers, as this exposure can significantly enhance their learning experience and practical skills

## 4 Discussion

The role of transplant nephrologists extends well beyond post‐operative management of pancreas transplant recipients. They are integral to candidate selection, perioperative decision‐making, immunosuppression management, and long‐term allograft monitoring. Fellowship training represents the foundational period for developing these competencies. It provides the structured clinical exposure necessary to build the knowledge base and clinical judgment required to independently manage this complex patient population. Without adequate training during fellowship, nephrologists are unlikely to acquire sufficient expertise later in their careers. The downstream consequences of inadequate fellowship training are therefore not confined to individual trainees; they propagate across the workforce, eroding the institutional expertise and critical mass necessary to sustain and develop successful pancreas transplant programs. This self‐reinforcing cycle highlights why addressing fellowship‐level training gaps is a necessary upstream intervention to expand pancreas transplant access and reverse declining national volumes.

This national survey highlights critical gaps in pancreas transplant training among transplant nephrology fellows. Overall, deficiencies in pancreas transplant training were largely attributed to low procedural volume, with a substantial proportion of programs (44%) reporting performing few or no pancreas transplants, thereby limiting opportunities for trainees to gain meaningful hands‐on experience. In contrast, programs performing 10 or more pancreas transplants annually were more likely to enable fellows to meet OPTN requirements for pancreas transplant medical directorship, underscoring the critical role of transplant volume in training and eligibility for leadership roles.

Low pancreas transplant volume fuels attrition of faculty expertise, resource allocation, and time allocated to pancreas transplant training for fellows at the institutional level, as reported by program directors in this survey. This calls for increasing focus on pancreas transplantation training, education, and research. Unfortunately, current transplant nephrology fellowship training programs do not require exposure to pancreas transplantation, further contributing to a decline in the pool of trained transplant nephrologists with the knowledge and skills required to manage this complex recipient population [2]. The inadequate training opportunities in pancreas transplantation during fellowship are perceived as contributing to the declining pancreas transplant volume in the US, despite advancements in outcomes [6, 7]. Although most programs provide didactic education and some clinical exposure, these offerings are often insufficient to meet the OPTN criteria to become a primary pancreas physician or to support the development of long‐term competence in pancreas transplant care.

Beyond pancreas transplant volume, a significant number of unfilled nephrology transplant fellowships each year threatens the pipeline of adequately trained pancreas transplant physicians. A recent survey of nephrology fellows by Sridhar et al. found that many trainees do not pursue transplant nephrology fellowship because of the additional training time required and other constraints [8]. The AST Fellows Task Force has proposed several recommendations to address these barriers to transplant nephrology training, which requires a sustained national effort for implementation [9].

Although 83% of transplant nephrology fellows receive some form of pancreas transplant education—primarily through didactic lectures‐ only 10% of programs reported offering formal training in pancreas allograft biopsies. Singh et al. reported substantial variation in pancreas allograft biopsy practices across U.S. pancreas transplant programs, as well as barriers to pancreas transplant utilization [10]. The near absence of structured training in pancreas transplant biopsies observed in our survey is a critical educational gap that primarily stems from a lack of faculty expertise and experience in pancreas transplants.

Program directors identified increasing pancreas transplant case volume and incorporating dedicated pancreas transplant rotations as the most effective strategies to address training barriers. Trainees from low‐volume pancreas transplant centers could complete elective rotations at those designated centers of excellence to enhance exposure to pancreas transplantation. In addition, there is a need to increase procedural exposure, foster interdisciplinary collaboration, especially with endocrinologists, and implement structured training pathways through standardized curricula or rotations at centers of excellence. These educational initiatives and policy decisions would strengthen the pancreas transplant workforce and ultimately improve access to pancreas transplantation for eligible patients. This recommendation aligns with prior recommendations from the AST‐sponsored pancreas workshop, held in conjunction with the American Transplant Congress (ATC) in Boston in June 2022, on the need to further develop formalized pancreas transplant training opportunities for both surgeons and nephrologists in medium‐ and high‐volume centers^2^. The Pancreas workshop also felt it would be both feasible and impactful to extend the OPTN qualifying period for pancreas certification to include a prespecified early faculty experience period to enable the acquisition of the case numbers required to serve as primary physicians and surgeons.

Moreover, the findings from this survey mirror those reported in the transplant surgery training literature, suggesting structural rather than discipline‐specific barriers. Fewer general surgery residents are pursuing transplant surgery training, making this fellowship one of the least competitive and sought‐after surgical fellowships for US‐trained residents [11]. Similar to pancreas training for transplant nephrology fellows, declining volumes lead to fewer surgical training opportunities, which produces fewer surgeons competent in pancreas transplantation, thereby reducing the number of active programs and case volumes [2].

This study has several strengths, including a high response rate of 72% across transplant nephrology fellowship programs in the United States, enhancing the reliability and generalizability of the findings. The comprehensive scope of the survey allowed for a detailed assessment of pancreas transplant training practices and barriers. However, the study is limited by potential response and recall biases inherent to self‐reported survey data. Additionally, non‐responder bias could affect the findings. The cross‐sectional design limits causal inferences and does not capture changes over time. Furthermore, the study lacks verification of actual fellow case logs, potentially leading to discrepancies between reported and actual training. It also focuses solely on program directors' perspectives, without insights from fellows. Lastly, the lack of linkage to post‐graduation outcomes limits the assessment of how training practices affect patient outcomes.

In summary, this national survey reveals that pancreas transplant training in transplant nephrology fellowships is constrained by low procedural volume and limited structured clinical exposure, preventing many fellows from meeting OPTN requirements or achieving long‐term competency. Addressing these gaps is critical to strengthening the pancreas transplant workforce and improving patient access. Supporting Information

## Author contributions

All authors contributed equally to the manuscript.

## Funding

The authors have nothing to report.

## Conflicts of Interest

The authors of this manuscript have no conflicts of interest

## Supporting information




**Supporting Information**: ctr70674‐sup‐0001‐DataCollectionForm.docx

## Data Availability

Data sharing not applicable to this article as no datasets were generated or analyzed during the current study.
