# Supplementary material for: Pancreas Transplant Training in U.S. Transplant Nephrology Fellowships: National Survey of Current Barriers and Potential Solutions
Source: Clin Transplant. 2026 Sep 9;40(9):e70674. doi: 10.1111/ctr.70674 (PMC13559214; doi:10.1111/ctr.70674)
Supplement: Supplementary file 1 [file CTR-40-e70674-s001.docx]

*Confidential*

**Survey for Transplant Nephrology Program Fellowship** *Page 1*

**Directors: Assessing Pancreas Transplant Training,**

**Barriers and Solutions**

Please complete the survey below. Thank you!

**Section 1: Demographic Information**

What is your program's UNOS center code?

How many transplant fellowship spots does your program offer per year?

In the past 3 years, how many times has your program failed to fill one or more (if you have >1 spot) of

your transplant fellowship positions?

How many pancreas transplants (including SPK, PAK, Pancreas alone) does your center perform in a year?

**Section 2: Pancreas Transplant Training**

Does your program enable fellows to meet the OPTN requirements to become a pancreas program medical director? (fellow must be involved in primary care of

8 or more newly transplanted pancreas recipients and followed them for a minimum of 3 months during the fellowship period)?

In the past 3 years, how many fellows from your program have met the OPTN requirements to become eligible for a pancreas program medical director position?

Do you have a dedicated pancreas transplant surgeon and/or physician to advocate for pancreas transplant at your program?

Does your center get endocrinology referrals of patients for pancreas transplants (including SPK, PAK, Pancreas alone)?

Do you offer any formal education or training of fellows specifically related to pancreas transplantation?

__________________________________

1 2 3

4 or more

0 times 1 time 2 times 3 times

≤5 6-9

10-20 >20

Yes

We don't have required pancreas transplant volume We don't do pancreas transplants at our center

0 1 2

3 or more

Not applicable (we do not have a pancreas transplant program)

Yes No

Yes No

Yes No

[projectredcap.org](https://projectredcap.org)

*Confidential*

*Page 2*

If yes, in what forms do you offer education (select all that apply):

If you chose "others" in the previous question, please specify

How would you rate the current level of pancreas transplant training provided to your fellows?

Lectures

Direct post-transplant patient care/management Participation of fellow in evaluating potential candidate and in selection committee meetings Pancreas transplant biopsy

Observing pancreas procurements and pancreas transplant surgeries

Others

__________________________________________

Excellent Good Adequate Inadequate Poor

**Section 3: Barriers to dedicated pancreas training and proposed solutions**

**Please rank the following barriers in order of their impact on providing adequate pancreas transplant training, with 1 being the most impactful and 7 being the least impactful:**

1	2	3	4	5	6	7

Limited number of pancreas transplants performed at the center

Lack of faculty expertise or availability

Insufficient time within the fellowship program

Competing priorities with other types of transplant training (e.g., kidney, liver)

Limited access to specialized educational resources

Institutional support and resources

Other

To what extent do you feel that these barriers impact the overall quality of pancreas transplant training

for transplant nephrology fellows in your program?

Significantly Moderately Slightly

Not at all

[projectredcap.org](https://projectredcap.org)

*Confidential*

*Page 3*

**Please rank the following strategies in order of their effectiveness in enhancing pancreas transplant training at your institution, with 1 being the most effective and 7 being the least effective:**

1	2	3	4	5	6	7

Increase the number of pancreas transplant cases for fellows

Incorporate dedicated pancreas transplant rotations

Provide more hands-on workshops or simulation training

Increase interdisciplinary collaboration with other transplant specialties

Develop and distribute comprehensive educational materials (e.g., manuals, online courses)

Invite external experts to provide lectures or workshops

Increase institutional support and funding for pancreas transplant training

How open would you be to implementing a standardized national curriculum or guidelines for pancreas transplant training?

Would you support the development of regional or national centers of excellence for pancreas transplant training, where fellows could rotate to gain more experience?

What additional resources or support would you need to overcome the barriers to effective pancreas transplant training at your institution?

Do you have any additional comments or suggestions regarding pancreas transplant training in transplant nephrology fellowship program?

Very open Somewhat open Neutral

Not open at all

Yes No Maybe

__________________________________

__________________________________

[projectredcap.org](https://projectredcap.org)
